# Plasma Proteome Profiling of Patients With In-stent Restenosis by Tandem Mass Tag-Based Quantitative Proteomics Approach

**DOI:** 10.3389/fcvm.2022.793405

**Published:** 2022-02-21

**Authors:** Jingyuan Hou, Qiaoting Deng, Sudong Liu, Xiaohong Qiu, Xunwei Deng, Wei Zhong, Zhixiong Zhong

**Affiliations:** ^1^Meizhou Academy of Medical Sciences Cardiovascular Disease Research Institute, Meizhou People's Hospital, Meizhou, China; ^2^Guangdong Provincial Key Laboratory of Precision Medicine and Clinical Translational Research of Hakka Population, Meizhou, China; ^3^Guangdong Provincial Engineering and Technology Research Center for Molecular Diagnostics of Cardiovascular Diseases, Meizhou, China; ^4^Guangdong Provincial Engineering and Technological Research Center for Clinical Molecular Diagnosis and Antibody Drugs, Meizhou, China; ^5^Center for Cardiovascular Diseases, Meizhou People's Hospital, Meizhou, China

**Keywords:** plasma, in-stent restenosis, quantitative proteomics, protein profiles, TMT, LC-MS/MS

## Abstract

**Background:**

Despite the widespread application of new drug-eluting stents, a considerable portion of patients experience in-stent restenosis (ISR). To date, the pathophysiologic mechanisms of ISR remain poorly understood.

**Methods:**

In this study, we collected plasma samples from ISR patients (*n* = 29) and non-ISR patients (*n* = 36) after drug-eluting stent implantation, as well as from healthy controls (HCs) (*n* = 32). Our goal was to investigate differences in plasma protein profiles using tandem mass tag (TMT) labeling coupled with liquid chromatography and tandem mass spectrometry. The proteomic data were validated by enzyme-linked immunosorbent assay (ELISA). Bioinformatic analyses were conducted to analyze potential pathways and protein-protein interaction (PPI) involved in ISR.

**Results:**

A total of 1,696 proteins were identified, of which 278 differed in protein abundance between non-ISR and HCs, 497 between ISR and HCs, and 387 between ISR and non-ISR, respectively. Bioinformatic analyses, including Gene Ontology (GO), Kyoto Encyclopedia of Genes and Genomes (KEGG) and PPI, further demonstrated that differentially abundant proteins between ISR and non-ISR are involved in several crucial biological processes and signaling pathways, such as focal adhesion, platelet activation, Rap1 signaling, regulation of actin cytoskeleton, and cholesterol metabolism. Among the identified differentially abundant proteins in ISR, 170 were increased in abundance relative to both non-ISR patients and HCs. Some of these proteins were identified to have critical functions for atherosclerosis development and might be involved in ISR pathology. Among these proteins, 3 proteins with increased abundance including fetuin-B, apolipoprotein C-III (APOC3), and cholesteryl ester transfer protein (CETP) were confirmed by ELISA.

**Conclusions:**

This is the first study provided a comprehensive proteomic profile to understand ISR pathology, which may help identify early diagnostic biomarkers and therapeutic targets.

## Introduction

Cardiovascular diseases are one of the major contributors to mortality and morbidity worldwide (1). Although percutaneous coronary intervention (PCI) is one of the most effective treatments for coronary artery disease, PCI with coronary stent implantation often leads to in-stent restenosis (ISR) (2). The risk of ISR is lower with newer-generation drug-eluting stents, but it continues to occur in 5–10% of patients undergoing PCI (3, 4). Furthermore, as the global population ages, the number of patients receiving stents has increased dramatically, leading to a concurrent rise in ISR incidence (5). Until recently, the optimal therapeutic strategy for ISR remains undefined and the underlying molecular mechanism involved in this process has not been fully understood. Hence, it is urgently needed to explore the molecular pathophysiology of ISR in-depth and to seek reliable biomarkers that can facilitate earlier diagnosis and aid efforts to treat and even prevent the condition.

Proteins play a vital role in mediating the functional regulation and signaling cascades, and their perturbation is closely linked to many pathological states (6). Plasma proteins are an easily accessible source of biomarkers that can reveal systemic alterations that lead to disease (7). In recent years, with the rapid development of mass spectrometry (MS) offering high resolution and fast scanning, proteomics has emerged as an indispensable analytical tool to study changes in protein abundances at the cellular level under various physiological or pathological conditions. The analysis of global protein abundances of samples from serum, plasma and other tissues can uncover novel protein markers for effective diagnosis and therapy (8). In fact, proteomics has identified several diagnostic and therapeutic biomarkers for oncological (9), neurological (10), autoimmune (11), and cardiovascular diseases (12–14). However, to date, the proteome of ISR has not been investigated.

Tandem mass tag (TMT)-based quantitative proteomics is a powerful proteomics technique with high sensitivity, accuracy, and reproducibility for the characterization and quantification of proteome dynamics (11). In combination with multidimensional liquid chromatography and tandem mass spectrometry (LC-MS/MS), the technology can simultaneously quantify up to 10 protein samples in a single analysis using a family of isobaric isotope tags (15). TMT is suitable for exploratory studies of the pathogenic mechanisms and pathophysiology of diseases.

Therefore, in this study, we collected plasma samples from ISR and non-ISR patients who received drug-eluting stents and healthy controls (HCs). We applied TMT-based quantitative proteomic analysis for the first time to elucidate the global plasma protein changes between ISR patients and the other two groups. In addition, we confirmed the certain differentially abundant proteins by enzyme-linked immunosorbent assay (ELISA), and we applied bioinformatic techniques to investigate their potential roles in ISR. Our findings may contribute to a better understanding of the pathogenesis of ISR and facilitate the identification of new potential biomarkers or therapeutic targets in the condition.

## Materials and Methods

### Study Design and Participants

This was a single-center retrospective study, conducted at Meizhou People's hospital, which is a large tertiary hospital with over 3,000 inpatient beds. Between October 2016 and April 2020, consecutive patients who received coronary artery drug-eluting stent implantation for the first time at the Department of Cardiology of Meizhou People's Hospital were prospectively recruited in our study. All patients received PCI and drug-eluting stent implantation according to standard guidelines. Drug-eluting stents were selected by the physician's discretion at the time of implantation and included everolimus-eluting stents (Taxus, Boston Scientific, Marlborough, MA), zotarolimus-eluting stents (Resolute integrity, Medtronic, Santa Rosa, CA) and rapamycin-eluting stents (Firebird2^TM^, Microport Inc. Shanghai, China). All patients who underwent successful PCI received a dual antiplatelet therapy consisting of 100 mg aspirin and 75 mg clopidogrel daily for at least 12 months. Patients returned for follow-up coronary angiography ranging from 6 to 12 months. The reasons for follow-up angiography were: (1) patients with elevated troponin blood level and either diagnostic electrocardiogram changes or ischemic symptoms during this time period. (2) patients without clinical symptoms during the follow-up period, coronary angiography was electively performed as follow-up examination at 12 months. ISR was defined as the presence of ≥50% angiographic diameter stenosis within or immediately adjacent (within 5 mm) of the implanted stent at follow-up angiography. Finally, 65 patients were enrolled and classified into the ISR group (*n* = 29) or non-ISR group (*n* = 36) based on the restenosis status, as confirmed by cardiologists.

Patients were included in this study if they (1) were older than 18 years, (2) received PCI and drug-eluting stent implantation, and (3) provided written informed consent. Patients were excluded if they had (1) a history of PCI and repeated stent implantation, (2) congenital heart disease or valvular disease, (3) current infection or chronic inflammatory disease, (4) malignancy or autoimmune disorder, or (5) severe hepatic failure or renal dysfunction. During the patient recruitment period, 32 outpatients who underwent health examinations at our hospital and had no discernible evidence of disease were selected as HCs. The study protocol was approved by the Institutional Review Board of the Meizhou People's Hospital (approval number: IRB-2019-C-66) and adhered to the principles of the Declaration of Helsinki. Furthermore, all participants signed a written informed consent prior to entering the study.

### Sample Collection and Processing

Fasting blood samples (5 mL) from patients prior to the follow-up coronary angiography and healthy individuals were drawn into BD Vacutainer® tubes (BD, Singapore) in the morning (16). The plasma was separated by centrifugation at 3,000 × *g* for 20 min at 4°C. Plasma samples in the supernatants were immediately dispensed into sterile Eppendorf tubes (Eppendorf AG, Hamburg, Germany) and stored as aliquots at −80°C until further processing.

### Protein Extraction and Trypsin Digestion

Sample pooling is a commonly used strategy to reduce the influence of inter-individual variation on candidate target selection in proteomic studies (17). Therefore, 27 plasma samples from the three groups (ISR, non-ISR, HCs; nine samples per group) were used for TMT proteomics analysis, where each sample was a pool from three randomly selected individuals. TMT analysis was performed at the Beijing Genomics Institute (BGI, Shenzhen, China).

**Figure 1:**
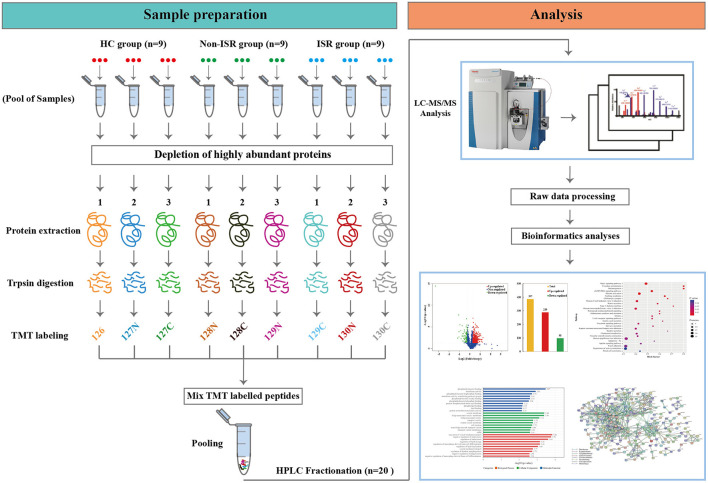
Scheme of the experimental design based on tandem mass tag (TMT) labeling combined with liquid chromatography and tandem mass spectrometry (LC-MS/MS). ISR, in-stent restenosis; HC, healthy controls.

The analytical flowchart is depicted in Figure 1. Briefly, the ProteoMiner Protein Enrichment Kit (Bio-rad Laboratories, Hercules, CA, USA) was utilized according to the manufacturer's instructions to deplete highly abundant proteins (18). Each tube was loaded with 900 μL of 0.22 μm-filtered sample and incubated for 2 h at room temperature, followed by addition of 100 μL of 1 M sodium citrate and 20 mM of 4-(2-hydroxyethyl)-1-piperazineethanesulfonic acid (pH 7.4). No bead aggregation was observed. The proteins were desorbed using a two-step elution. First, the beads were incubated twice with 100 μL of the kit elution reagent [4 M urea, 1% (w/v) 3-(3-cholamidopropyl) dimethylammonio-1-propanesulfonate, 5% (v/v) acetic acid] for 15 min. Then, 100 μL of 6 M guanidine-HCl (pH 6.0) was added twice for 15 min. Four elution fractions from each column were collected, pooled, and stored at −80°C until further analysis. The final protein concentration and quality were determined by Bradford method using the Protein Assay Kit (Bio-Rad, Hercules, CA, USA) and 15% sodium dodecyl sulfate–polyacrylamide gel electrophoresis.

Total proteins (100 μg) from each sample were dissolved in 0.5 M triethylammonium bicarbonate (TEAB) at pH 8.0 (Applied Biosystems, Milan, Italy). Trypsin Gold (Promega, Madison, WI, USA) was then added at a trypsin/protein mass ratio of 1:40 to digest the proteins at 37°C for 18 h. After trypsin digestion, tryptic peptides were treated with 0.5% (v/v) formic acid to terminate the enzymatic reaction, which was desalted over a Strata X solid-phase extraction C18 column (Phenomenex, CA, USA). Eluates were vacuum-dried.

### TMT Labeling

The peptides were dissolved in TEAB and then labeled with the TMT 10-plexed label reagent set (Thermo Fisher Scientific, Waltham, MA, USA) according to the manufacturer's protocol. Briefly, each tube of TMT reagent (0.8 mg) was thawed and reconstituted in 41 μL of acetonitrile (ACN), and the peptides were dissolved in 30 μL of 0.1 M TEAB solution. Then, 100 μg of peptide (26.7 μL) and 41 μL of TMT reagent were rapidly mixed and allowed to stand at room temperature for 2 h to achieve complete labeling. Next, the labeled peptide mixtures were pooled, desalted, and dried via vacuum centrifugation. A total of nine pooled samples (three biological replicates of each group) were labeled with the TMT tags as follows: HC samples were labeled with TMT tags 126, 127N, and 127C; non-ISR samples were labeled with TMT tags 128N, 128C, and 129N; while ISR samples were labeled with TMT tags 129C, 130N, and 130C.

### Separation of Peptides and LC-MS/MS Analysis

Strong cation exchange (SCX) chromatography was performed on a Shimadzu LC-20AB HPLC Pump system (Shimadzu, Nakagyo-ku, Kyoto, Japan) coupled with a high pH reverse phase column. Briefly, the TMT-labeled peptides were reconstituted with mobile phase A (5% ACN, 95% water, pH adjusted to 9.8 with ammonia) into a 2 mL volume and loaded onto a Ultremex SCX column containing 5-μm particles (Gemini C18, 4.6 mm × 250 mm, Phenomenex, CA, USA). Peptide samples were subsequently eluted at a flow rate of 1 mL/min using the following gradient of mobile phase B (5% water, 95% ACN, pH adjusted to 9.8 with ammonia): 0–10 min, 5% B; 10–50 min, 5–35% B; and 50–51 min, 35–95% B. The system was then maintained in 95% B for 3 min, which was decreased to 5% within 1 min, and then the column was equilibrated in 5% B for 10 min. The peptides were monitored by measuring absorbance at 214 nm, and fractions were collected every min during the linear elution period. Finally, the eluted peptides were pooled as 20 fractions and vacuum-dried (19).

All vacuum-dried fractions were resuspended with mobile phase A (2% ACN, 0.1% formic acid (FA) and centrifuged at 20,000 × *g* for 10 min. Subsequently, 10 μL of supernatant was loaded on an HPLC system (Thermo Scientific™ UltiMate™ 3,000 UHPLC, Thermo Scientific, Chelmsford, MA, USA) equipped with a trap and an analytical column. The samples were loaded on a trap column to be enriched and desalted. Then, the peptides were separated at 5 μL/min for 8 min, and then eluted into a 25-cm analytical column [C18 Acclaim PepMap 100, 75 μm (inner diameter) × 25 cm, 3 μm particles, Dionex, Thermal Scientific, Chelmsford, MA, USA] at a flow rate of 300 nL/min. A linear gradient of buffer B (98% ACN, 0.1% formic acid) was applied: 0–40 min, 5–25% B; 40–45 min, 25–35% B; 45–47 min, 35–80% B; 47–49 min, 80% B. The percentage of B was then reduced to 5% within 1 min and maintained there for 6 min to re-equilibrate the column.

The peptides separated from HPLC were ionized by a nano-electrospray ionization source and then subjected to tandem mass spectrometry with a Q Exactive HF X (Thermo Fisher Scientific) under the data-dependent acquisition (DDA) mode. All tandem mass spectra were generated using the higher-energy collision dissociation (HCD) approach. The parameters for MS analysis were as follows: electrospray voltage, 2.0 kV; precursor scan range, 350–1,500 m/z at a resolution of 60,000 in Orbitrap; MS/MS fragment scan range, >100 m/z at a resolution of 15,000 in HCD mode; normalized collision energy setting, 30%; dynamic exclusion time, 30 s; automatic gain control for full MS target and MS2 target, level 1 3E6 and level 2 1E5, respectively; number of MS/MS scans following one MS scan, 20 most abundant precursor ions above a threshold ion count of 10,000.

### Protein Identification and Quantification

Mascot software (version 2.3.02; Matrix Science, Boston, MA, USA) was used offline to identify proteins from the processed MS data. Raw MS/MS data were converted into mascot generic format (MGF) using Proteome Discover 1.4 (Thermo Fisher Scientific, Waltham, USA) before analysis. The MGF files mainly contained information about the secondary MS spectrum, and then the converted files were searched by the Mascot search engine against the UniProt Homo sapiens 20358-20190322.fasta protein database (downloaded on May 22nd, 2019, with 20,358 protein sequences) concatenated with reverse decoy database for protein identification. The following parameters were used for database searches: monoisotopic mass accuracy; MS/MS fragment ion mass tolerance, ± 0.05 Da; tolerance of one missed cleavage in the trypsin digests; peptide mass tolerance, 20 ppm; variable modifications of oxidation (M); fixed modifications of carbamidomethyl (C), TMT10plex (N-terminal) and TMT10plex (K). The default settings were used for all other parameters. Each protein was identified on the basis of at least one unique peptide.

For protein quantification, we used the iQuant software (20) (version 2.2.1) independently developed by BGI (Shenzhen, China), which integrates the Mascot Percolator algorithm (21) using machine learning algorithms to automatically re-score database search results, thereby improving the identification rate. First, filtering with a 1% false discovery rate (FDR ≤ 0.01) was applied to peptide-spectrum matches to obtain significantly identified spectra and peptide lists. Then, peptides were assembled according to the “parsimony principle” to produce a series of proteomes, which were filtered for 1% FDR at the protein level. Proteins were selected with the picked protein FDR strategy (22) in iQuant through the following steps: protein filtration, report-group tag-purity correction, quantitative value normalization, missing value completion, protein quantitative-value calculation, statistical test analysis, and final result display. The proteins with an average fold-change in abundance >1.20 or <0.83 (*p* < 0.05) were considered differentially abundant proteins.

### Bioinformatic Analysis

Functional analysis of differentially abundant proteins was conducted using Gene Ontology (GO) annotation (http://www.geneontology.org/) and proteins were categorized in terms of biological processes, molecular functions, and cellular localization. Differentially abundant proteins were further assigned to functional pathways according to the Kyoto Encyclopedia of Genes and Genomes (KEGG) database (http://www.genome.jp/kegg/pathway.html). GO terms and KEGG pathways with a *p*-value < 0.05 were considered significantly enriched. Protein-protein interaction (PPI) networks were analyzed using the online STRING database version 11.0 (https://string-db.org/), and the minimum required interaction score was set at the highest confidence of 0.9.

### ELISA Validation of Protein Expression

To validate findings from the TMT-based proteomic profiling, three differentially abundant proteins among the HC, non-ISR, and ISR groups were selected and assayed by ELISA. ELISA kits for plasma fetuin-B and apolipoprotein C-III (APOC3) were purchased from Abcam (Cambridge, UK), while a kit for cholesteryl ester transfer protein (CETP) was purchased from Cusabio Biotech (Wuhan, China). Plasma protein levels were measured according to the manufacturers' protocols. The optical density at 450 nm was measured using a Varioskan Flash ELISA reader (Thermo Fisher Scientific, San Jose, CA, USA).

### Statistical Analysis

Statistical analyses were performed using the Statistical Program for Social Sciences software 20.0 (IBM, Armonk, NY, USA). Continuous variables were reported as the mean ± standard deviation (SD), meanwhile One-way analysis of variance (one-way ANOVA) was used for the comparison among multiple groups, and LSD-*t* test was used for pairwise comparison between groups. Categorical variables were presented as frequencies and percentages, while Chi-square test was used to compare differences between groups. *P* < 0.05 was considered statistically significant.

## Results

### Characteristics of the Study Population

A total of 97 plasma samples were obtained from 29 patients who experienced ISR after drug-eluting stent implantation, 36 patients who did not experience ISR after stent implantation, and 32 HCs. Nine subjects of each group were randomly selected for proteomics analysis to identify differentially abundant proteins. The remaining samples were utilized for validation of the TMT proteomic analysis results. The detailed demographic and clinical characteristics of the subjects are presented in Table 1.

**Table 1:** Demographic and clinical characteristics of the study population.

**Characteristic**	**Proteomic analysis**				**Verification study**			
	**ISR** **(*n* = 9)**	**Non-ISR** **(*n* = 9)**	**HC** **(*n* = 9)**	***P*-value**	**ISR** **(*n* = 20)**	**Non-ISR** **(*n* = 27)**	**HC** **(*n* = 23)**	***P*-value**
Age (years)	65.78 ± 11.27	66.22 ± 7.26	63.33 ± 4.33	0.723	65.60 ± 12.05	68.11 ± 11.97	61.26 ± 7.59	0.086
Males	6 (66.7)	6 (66.7)	6 (66.7)	1.000	15 (75.0)	18 (66.7)	9 (39.1)	0.038
Current smokers	2 (22.2)	1 (11.1)	1 (11.1)	0.746	2 (10.0)	2 (7.4)	2 (8.7)	0.952
Diabetes mellitus	1 (11.1)	1 (11.1)	1 (11.1)	1.000	12 (60.0)	8 (29.6)	2 (8.7)	0.001
Hypertension	7 (77.8)	5 (55.6)	2 (22.2)	0.060	17 (85.0)	18 (66.7)	10 (43.5)	0.017
Hyperlipidemia	2 (22.2)	2 (22.2)	2 (22.2)	1.000	6 (30.0)	6 (22.2)	4 (17.4)	0.614
Stent diameter (mm)	3.01 ± 0.23	3.13 ± 0.32	0	0.383	3.07 ± 0.32	2.97 ± 0.75	0	0.649
Total stent length (mm)	24.53 ± 8.45	22.50 ± 7.04	0	0.587	23.86 ± 5.30	24.88 ± 6.46	0	0.568
**Current medications**								
Aspirin	8 (88.9)	9 (100.0)	1 (11.1)	<0.001	20 (100.0)	24 (88.9)	8 (34.8)	<0.001
ACE/ARB blocker	7 (77.8)	8 (88.9)	3 (33.3)	0.030	19 (95.0)	23 (85.2)	14 (60.9)	0.014
Beta-blocker	8 (88.9)	7 (77.8)	5 (55.6)	0.259	18 (90.0)	25 (92.6)	14 (60.9)	0.008
Calcium blocker	1 (11.1)	1 (11.1)	3 (33.3)	0.375	4 (20.0)	3 (11.1)	4 (17.4)	0.684
Statins	9 (100.0)	9 (100.0)	3 (33.3)	<0.001	20 (100.0)	25 (92.6)	12 (52.2)	<0.001
Creatinine (μmol/L)	96.67 ± 35.27	98.22 ± 24.80	83.22 ± 12.18	0.414	113.58 ± 45.64	99.09 ± 28.53	104.80 ± 144.85	0.857
Triglyceride (mmol/L)	1.85 ± 0.82	2.07 ± 2.19	1.59 ± 0.52	0.767	1.93 ± 2.28	1.66 ± 0.98	2.24 ± 2.07	0.532
Total cholesterol (mmol/L)	4.21 ± 1.18	4.53 ± 0.88	4.87 ± 0.99	0.413	4.63 ± 0.92	4.30 ± 1.54	5.13 ± 0.92	0.058
LDL-C (mmol/L)	2.40 ± 0.79	2.31 ± 0.66	2.76 ± 0.80	0.426	2.42 ± 0.72	2.16 ± 1.03	2.57 ± 0.68	0.220
HDL-C (mmol/L)	1.03 ± 0.15	1.36 ± 0.53	1.29 ± 0.30	0.148	1.14 ± 0.27	1.17 ± 0.42	1.20 ± 0.28	0.807
Glucose (mmol/L)	6.34 ± 2.70	4.84 ± 0.98	5.72 ± 2.84	0.403	6.21 ± 2.26	5.96 ± 1.81	5.34 ± 1.40	0.290

*Data was presented as mean ± SD or n (%) according to variable category. ANOVA analysis or Chi-square test was used to compare differences between groups. ACE, angiotensin-converting enzymeinhibitor; ARB, Angiotensin receptor blocker; HC, healthy control; HDL-C, High-density lipoprotein cholesterol; ISR, In-stent restenosis; LDL-C, Low-density lipoprotein cholesterol*.

### Protein Identification

To improve the reliability of data, three biological replicates from each group were included in the TMT experiment (Figure 1), which generated a total of 697,591 MS/MS spectra, of which 33,404 were matched to known human spectra in the reference genomes using Mascot software (Matrix Science, London, UK; version 2.3.02). Among these, 30,011 were found to be unique spectra. In the end, 7,674 peptides (7,333 unique) and 1,696 proteins were identified based upon the criteria of FDR <1% and at least one unique peptide (Supplementary Tables 1–3).

**Figure 2:**
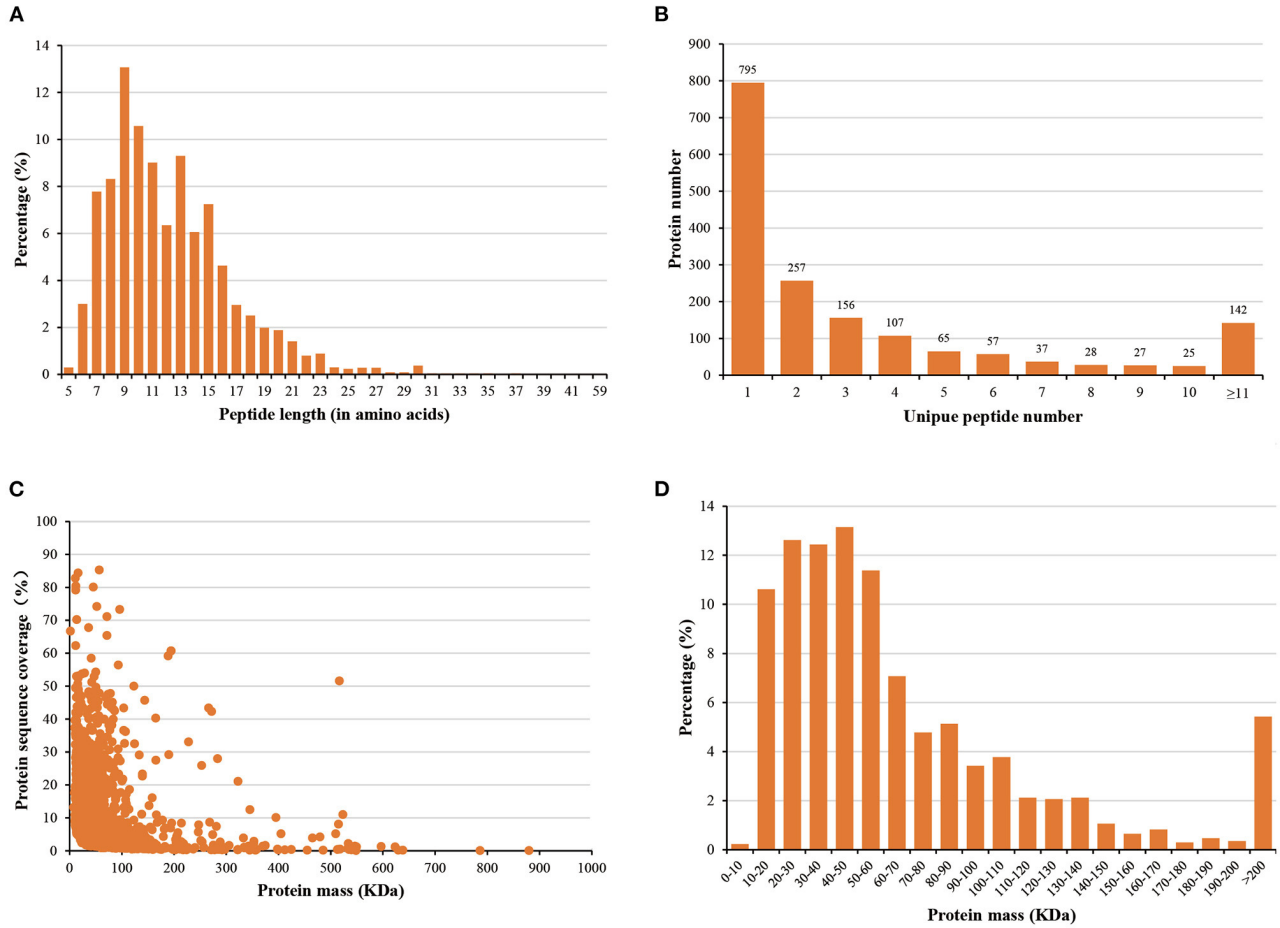
Distribution of **(A)** peptide lengths, **(B)** numbers of peptides, **(C)** protein sequence coverage, and **(D)** protein mass.

The statistical analysis showed that most of the peptides were between 6 and 23 amino acids long (Figure 2A). In addition, 901 (53.1%) of all identified proteins were identified based on at least two peptides per protein, while 1,497 (88.3%) were identified within 10 peptides (Figure 2B). We observed that the protein masses ranged from 1.37 to 879.61 kDa (Figure 2C), with more than 90.0% of the proteins having masses between 10 and 140 kDa (Figure 2D). These results suggested that the data we obtained was relatively high-quality and reliable.

### Analysis of Differentially Abundant Proteins

**Figure 3:**
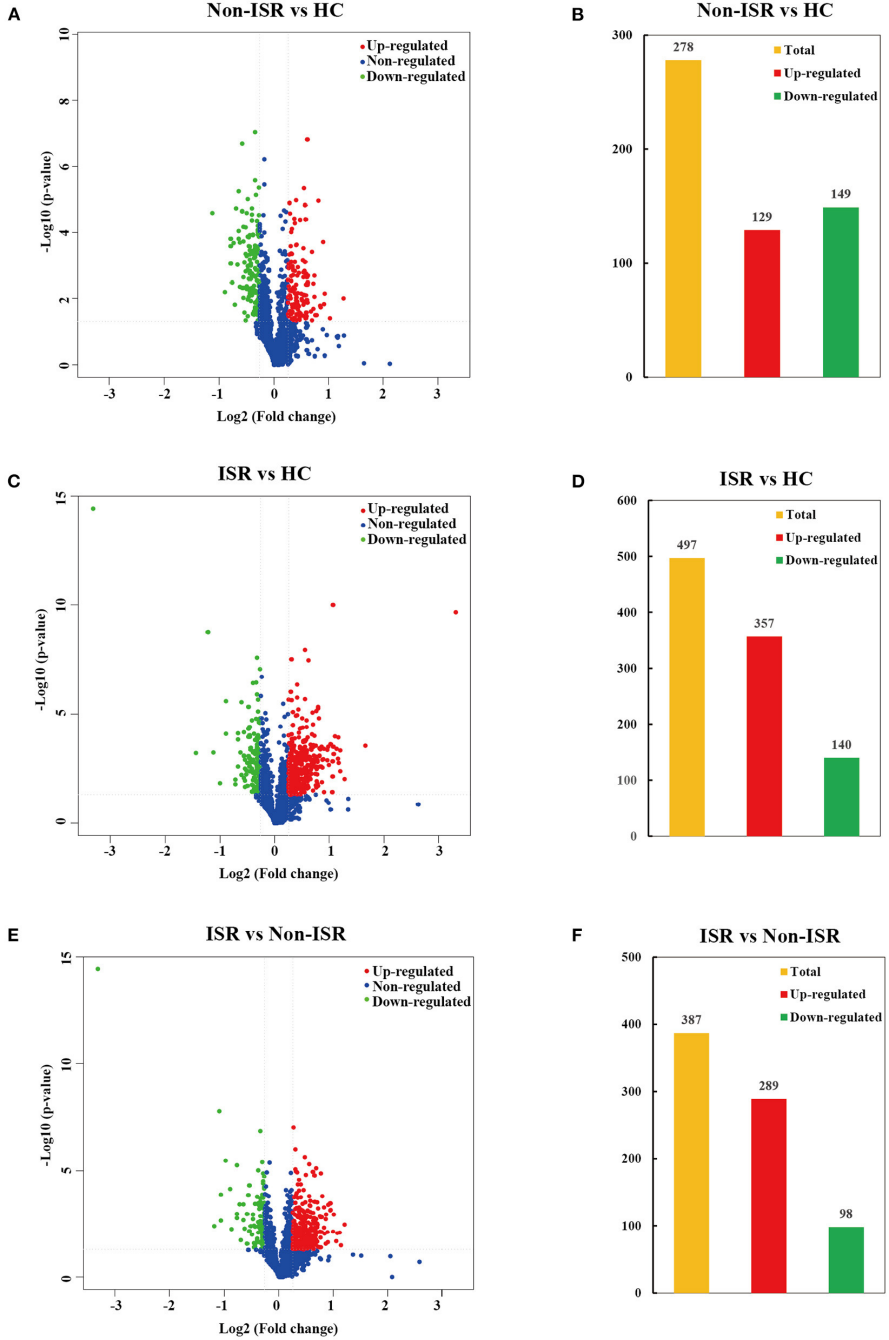
Volcano plots and histogram statistics of differentially abundant proteins between **(A,B)** non-ISR patients and HCs, **(C,D)** ISR patients and HCs, or **(E,F)** ISR and non-ISR patients. Increased abundance proteins are indicated in red (ratio > 1.20), while decreased abundance proteins are indicated in green (ratio < 0.83) with *p* < 0.05. The x-axis displays the log_2_ (fold change) for proteins, and the y-axis displays the corresponding log_10_-transformed *p*-values.

In order to comprehensively understand the plasma protein profiles in patients with ISR, we generated three sets of comparative proteomics data for ISR, non-ISR, and HC by the TMT labeling approach. Overall, 1,696 proteins were successfully identified by LC-MS/MS in the three groups (Supplementary Table 4). The significantly differential abundant proteins were screened by a cut-off threshold fixed at fold change >1.20 or <0.83 with a *p*-value < 0.05. Finally, a total of 278 proteins showed differences between the non-ISR and HC groups, with 129 increased abundance proteins and 149 decreased abundance proteins in the non-ISR group (Figures 3A,B; Supplementary Table 5). A total of 497 differentially abundant proteins were identified between the ISR and the HC groups; of these, 357 were increased in abundance and 140 decreased in abundance in ISR (Figures 3C,D; Supplementary Table 6). A total of 387 differentially abundant proteins were identified between the ISR and non-ISR groups; 289 of these were increased in abundance and 98 decreased in abundance in ISR (Figures 3E,F; Supplementary Table 7). It merits noting that the number of the differentially abundant proteins between ISR patients and HCs was the highest, followed by ISR and non-ISR comparison, and non-ISR and HCs comparison was the least. These results suggest that the plasma proteins in ISR patients are quite different from those in non-ISR patients and HCs, likely due to the pathophysiological process of ISR.

**Figure 4:**
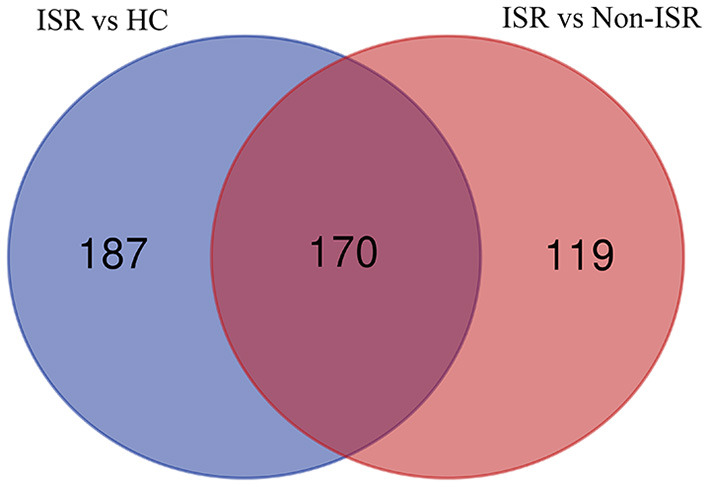
Venn diagram showing the overlap of increased abundance proteins between comparisons of ISR patients with HCs (purple), and of ISR with non-ISR patients (red).

Considering that increased abundance proteins may have more significant potential and convenience as diagnostic markers than decreased abundance ones, we further analyzed increased abundance proteins in ISR patients compared with non-ISR patients and HCs. The numbers of proteins with increased abundance and how they overlapped between both comparisons were depicted in a Venn diagram. Of the 476 increased abundance proteins in ISR patients, 170 overlapped (Figure 4; Supplementary Table 8). Some of these proteins have been shown to play essential roles in the development of atherosclerosis, such as low-density lipoprotein receptor-related protein 4 (LRP4) (23), fetuin-B (24), calponin-2 (25), and proteoglycan 4 (26).

### Functional Categorization of Differentially Abundant Proteins

**Figure 5:**
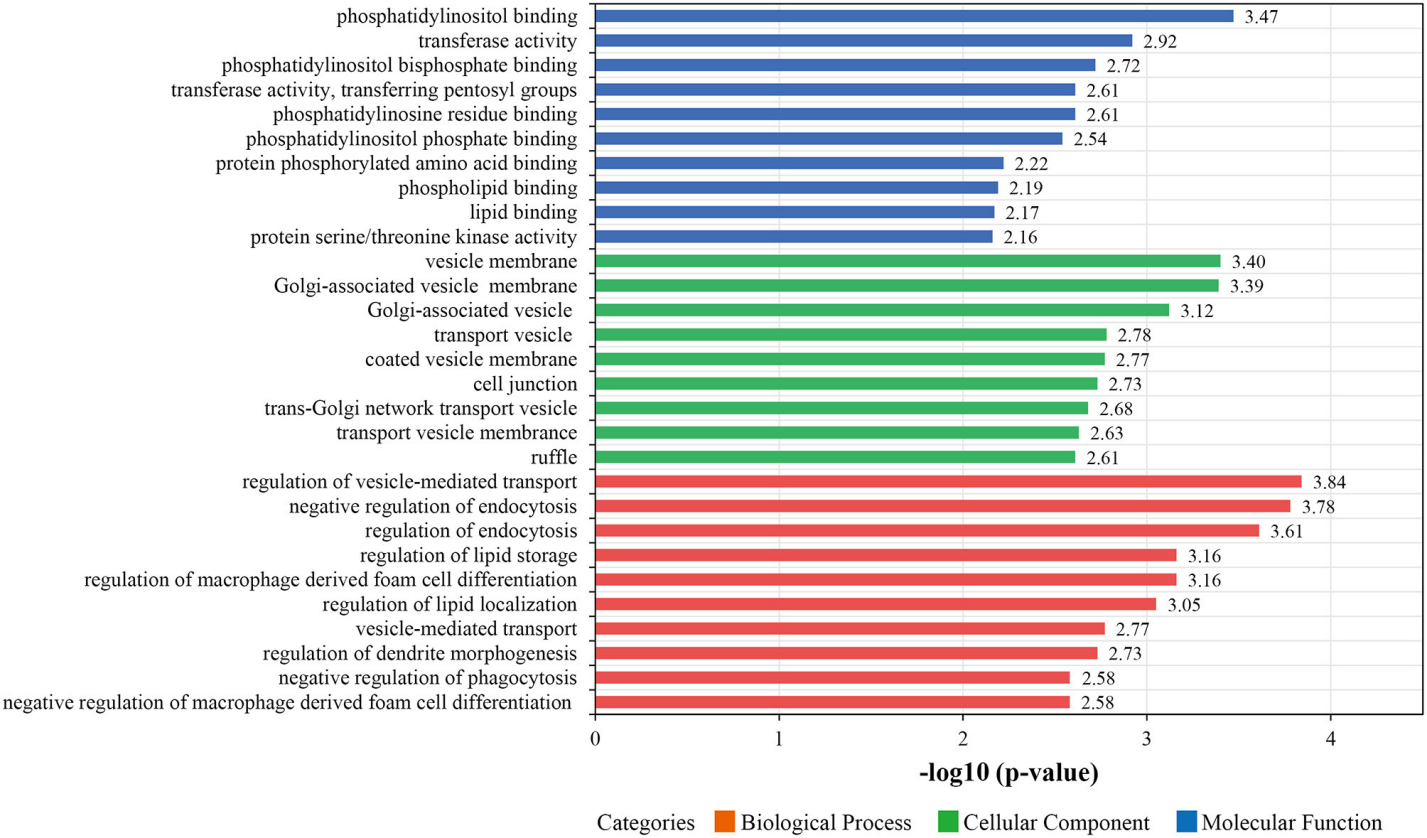
The top 10 most enriched Gene Ontology (GO) terms for differentially abundant proteins between the ISR and non-ISR groups. The ordinate displays the name of each GO term. The color implies the three main GO categories of molecular functions (blue), biological processes (red), and cellular components (green). Each bar denotes a GO term, and the length of the bar represents the enrichment significance [-log (*p*-values), *p* < 0.05].

In subsequent analyses, we focused on proteins that were changes in abundance levels during the pathological process of ISR. Based on GO analysis, the 387 with increased or decreased abundance between the ISR and non-ISR groups were categorized in terms of biological processes, cellular components, and molecular functions. The top 10 most enriched molecular functions, cellular components, and biological processes are shown in Figure 5 and Supplementary Table 9. In the classification of molecular functions, the significant top 10 enrichment items are phosphatidylinositol binding, transferase activity, phosphatidylinositol bisphosphate binding, phosphotyrosine residue binding, transferase activity, transferring pentosyl groups, phosphatidylinositol phosphate binding, protein phosphorylated amino acid binding, phospholipid binding, lipid binding and protein serine/threonine kinase activity. In the category of cellular components, the top 10 significant enrichment items are cytoplasmic vesicle membrane, vesicle membrane, Golgi-associated vesicle membrane, Golgi-associated vesicle, transport vesicle, coated vesicle membrane, cell junction, trans-Golgi network transport vesicle, transport vesicle membrane and ruffle. Moreover, regulation of vesicle-mediated transport, negative regulation of endocytosis, regulation of endocytosis, regulation of macrophage derived foam cell differentiation, regulation of lipid storage, regulation of lipid localization, vesicle-mediated transport, regulation of dendrite morphogenesis, negative regulation of macrophage derived foam cell differentiation and negative regulation of phagocytosis were the top 10 significantly rich terms in the biological processes.

**Figure 6:**
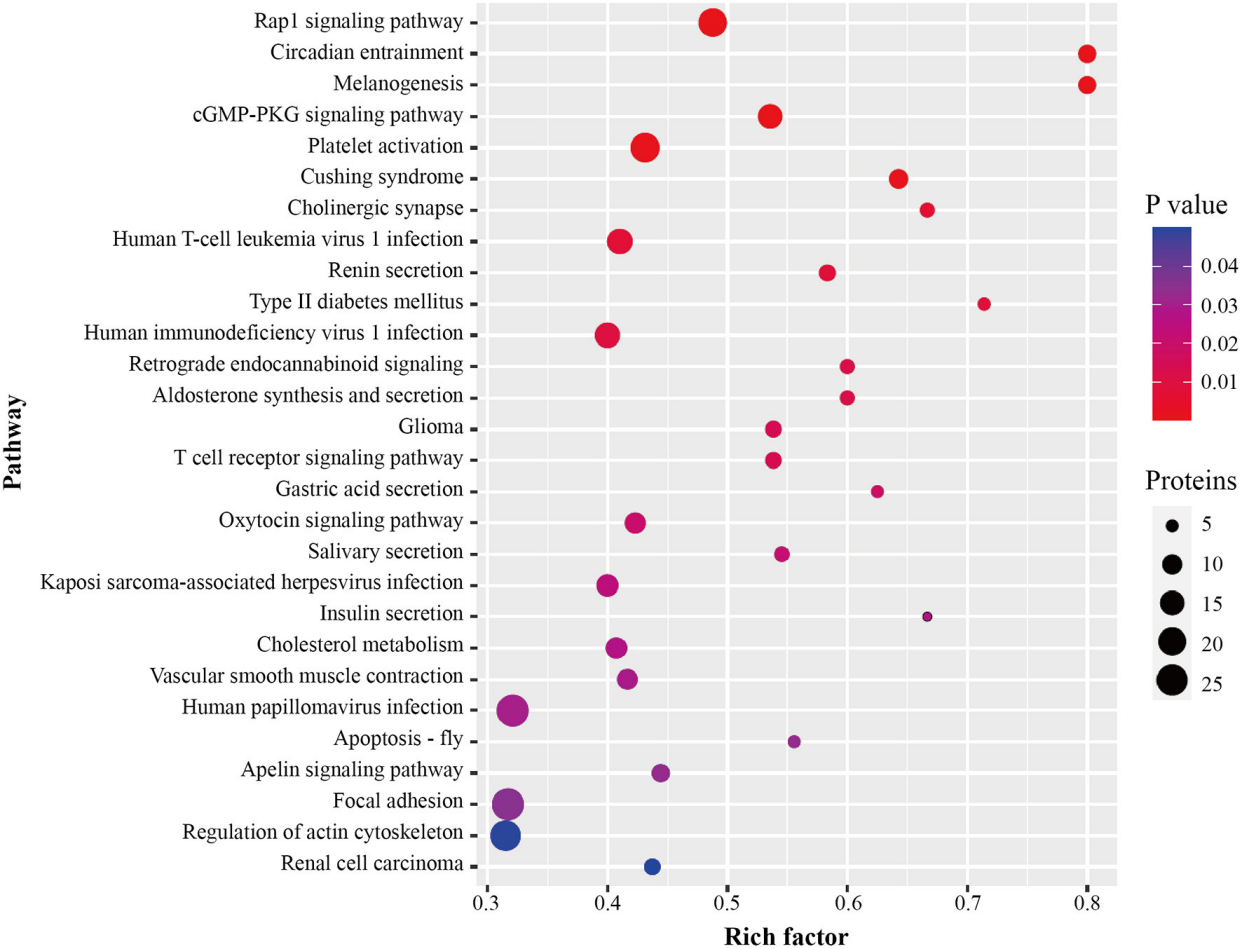
Pathway enrichment of differentially abundant proteins between ISR and non-ISR groups. The dot size represents the number of proteins involved in each KEGG pathway, and the color gradient corresponds to the *p*-value-based statistical significance.

After identification of plasma proteins significantly altered between the ISR and non-ISR groups, a KEGG pathway analysis was performed to investigate their potential biological functions. The dysregulated proteins mapped mainly onto 28 KEGG pathways (Figure 6; Supplementary Table 10). A total of 371 pathway proteins were enriched in the KEGG pathway enrichment analysis. Noteworthy, several of those pathways may be involved in the development of ISR: Rap1 signaling pathway, cGMP-PKG signaling pathway, platelet activation, T cell receptor signaling pathway, cholesterol metabolism, vascular smooth muscle contraction, focal adhesion, and regulation of actin cytoskeleton.

**Figure 7:**
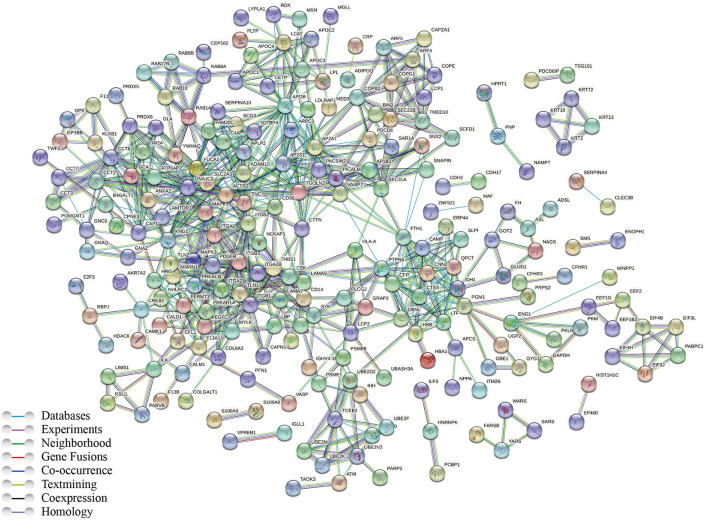
Network of protein interactions in ISR, generated using the STRING database. Lines are colored according to the type of association between the indicated proteins (see legend).

**Figure 8:**
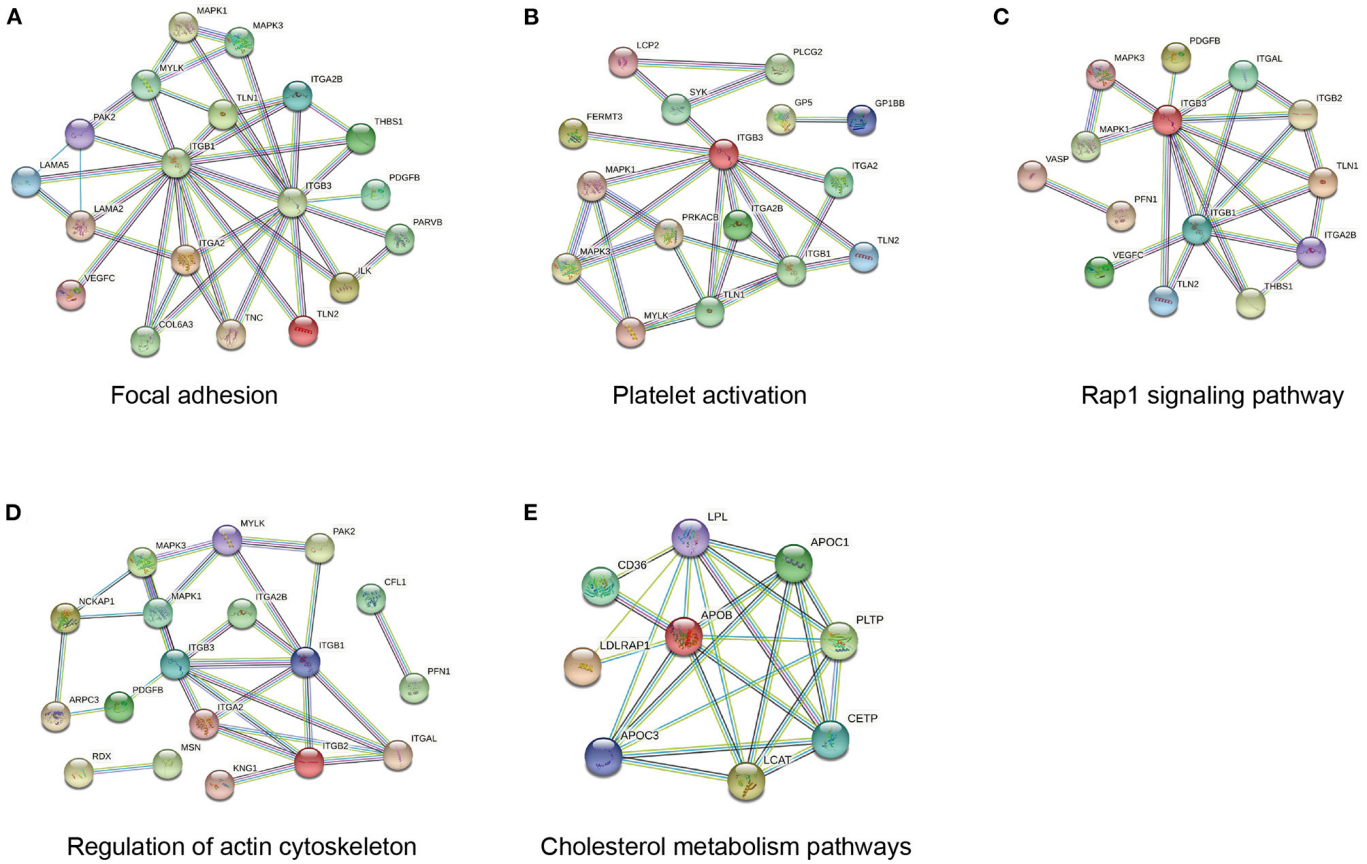
Network of protein interactions in ISR involved in **(A)** focal adhesion, **(B)** platelet activation, **(C)** Rap1 signaling pathway, **(D)** regulation of actin cytoskeleton, **(E)** cholesterol metabolism pathway.

To better understand the biological pathways involved in the mechanisms of ISR, we further used STRING database to construct interaction networks for the differentially abundant proteins between the ISR and non-ISR groups (Figure 7). The protein network analysis obtained with STRING database revealed that the majority of the proteins have had a strong correlation in terms of known and predicted protein-protein interactions. Among them, several crucial biological processes and signaling pathways related to atherosclerosis and cardiovascular disease have been previously reported, such as focal adhesion, platelet activation, Rap1 signaling pathway, regulation of actin cytoskeleton, and cholesterol metabolism pathway (Figure 8). These pathways and the interactions among the proteins involved may be critical in the occurrence and evolution of ISR and deserve our further attention.

### Validation of Differentially Abundant Proteins by ELISA

**Figure 9:**
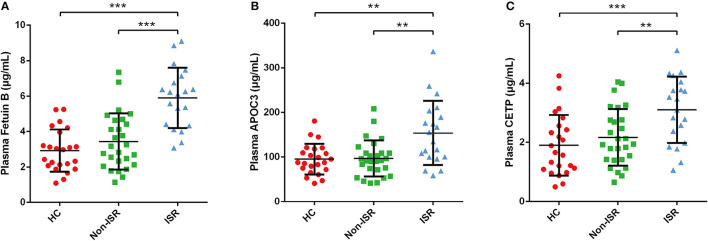
ELISA validation of differentially abundant plasma proteins in ISR. Plasma level of **(A)** Fetuin B, **(B)** APOC3, **(C)** CETP. Data came from 23 HCs, 20 ISR patients and 27 non-ISR patients. Data were expressed as mean ± SD. LSD-*t* test was used to compare differences between groups. Statistical significance was defined as **p < 0.01 and ***p < 0.001.

Based on the above analysis results, we preferentially selected the proteins increased in abundance in the ISR group for further verification by ELISA. Consistent with our proteomic analysis, the plasma levels of fetuin-B, APOC3, and CETP were significantly higher in the ISR group than in the non-ISR or HC groups (Figure 9).

## Discussion

Despite the use of contemporary drug-eluting stents, ISR remains a vexing clinical problem, affecting a considerable portion of patients undergoing PCI (2, 3). The pathophysiologic mechanism of ISR is not known, but it is currently thought that vascular inflammation, platelet activation, smooth muscle cell proliferation and migration, and extracellular matrix re-modeling are responsible for the neointimal hyperplasia causing a re-narrowing of the arterial lumen, which lead to the occurrence and evolution of ISR (27–29). Proteomics profiles the abundance and function of the protein on a global scale, providing a rapid and precise way to discover and identify differentially abundant proteins and to characterize certain disease states. To date, the proteome of ISR has not been previously undertaken.

In our study, for the first time, TMT proteomic analysis was employed to reveal the proteomic profile alterations in plasma samples from patients with ISR, and bioinformatic analyses of differentially abundant proteins was carried out. In the design of the proteomic studies, the procedures of sample collection and processes influence the composition of the complex samples, and hence perturbs the actual protein abundances. In particular, human plasma contains extraordinary diverse proteins and the ingestion or circadian rhythms may affect its secretion considerably and cause interferences (30). Therefore, in this study, blood samples were collected from fasting patients in the morning. The dynamic variations among the individual plasma samples are relatively large, and thus the pooled plasma samples strategy was adopted to reduce individual differences and enhance the confidence of this study (17). Additionally, highly abundant proteins present in the plasma samples can mask some low abundant proteins and cause loss of resolution in proteomic investigations (31). Considering this, in our study, the high abundance proteins albumin, and immunoglobulins were depleted from the pooled plasma samples with the commercial ProteoMiner kit (18), resulting in the enrichment and identification of medium and low abundant plasma proteins from plasma. Meanwhile, in order to quantify more peptides, the eluted TMT-labeled peptides were combined into 20 fractions and each fraction was analyzed by LC-MS/MS (19). Although the detectability of proteins with TMT can be limited by ion co-elution-induced ratio compression, its performance has robustly matured into sensitivity and dynamic range that makes it interesting for discovery of plasma biomarkers (32). To further improve the identification of plasma proteins, quantitative analysis of the peptides labeled with isobaric tags was performed with IQuant software (20). Following the optimization as mentioned above, a total of 1,696 plasma proteins were finally identified by using TMT-based quantitative proteomic analysis. The identification results revealed that the combined strategies performed at the protein and peptide levels were efficient and reliable for identifying proteins.

We showed a clear difference among the protein profiles of the ISR, non-ISR, and control groups: 387 candidate proteins with differential abundances were identified between ISR and non-ISR patients. After revealing the significantly dysregulated proteins, we performed bioinformatic analyses to extract functional information from the proteomic data. Differentially abundant proteins were involved mainly in the regulation of macrophage-derived foam cell differentiation, regulation of lipid storage, regulation of lipid localization, and negative regulation of macrophage-derived foam cell differentiation. It is consistent with the literature reported that disorder of lipid metabolism is closely associated with ISR in patients after coronary artery stent therapy, thus intensive treatment with statin is recommended to reduce the occurrence of in-stent restenosis (33, 34). Further functional category analysis and protein-protein interactions indicated that these proteins were enriched in several pathways such as Rap1 signaling, cGMP-PKG signaling, platelet activation, T cell receptor signaling, cholesterol metabolism, vascular smooth muscle contraction, focal adhesion, and regulation of actin cytoskeleton. Altogether, these biological processes and pathways mainly involve smooth muscle and endothelial cells, but also monocytes, macrophages, T cells, and platelets, which participate in vascular remodeling and contribute to the progression of atherosclerosis (35, 36). Our insights into the protein interaction networks and signaling pathways involved in ISR are consistent with suggestions about how ISR occurs, suggesting that our analysis is reliable and provides trustworthy candidates for further study. Indeed, several differentially abundant proteins we identified in this study, such as LRP4 (23), fetuin-B (24), calponin-2 (25), and proteoglycan 4 (26), have already been linked to atherosclerosis.

After reviewing a wealth of literature, we discovered potential clues between lipid profiles and ISR. A previous study identified very low-density lipoprotein cholesterol as an independent risk factor associated with ISR in diabetic patients (37). One observational cohort study provided evidence that the presence of remnant-like particle cholesterol is an independent risk factor for in-stent restenosis in diabetic patients (38). Another recent study found that a prediction model including abnormal total cholesterol and low-density lipoprotein cholesterol (LDL-C) levels could predict ISR in coronary artery disease patients after coronary DES implantation (39). Considering our previous research and interest in the relationship between lipids and cardiovascular diseases (40, 41), we herein used ELISA to validate three differentially abundant proteins involved in cholesterol metabolism: fetuin-B, APOC3, and CETP. As expected, the validation results were consistent with the proteomics results (Figure 9). Further studies on verifying these proteins may serve as potential biomarkers and provide information that could reveal potential physiological mechanisms of ISR.

Lipoprotein metabolism has been well-documented to play a prominent role in atherosclerotic cardiovascular disease by affecting lipid accumulation and atherosclerotic plaque formation (42, 43). Fetuin-B is a 380-residue glycoprotein and belongs to the cystatin superfamily of cysteine protease inhibitors, which is predominantly synthesized in the liver and secreted into the circulation. The liver plays a pivotal role in the regulation of systemic glucose and lipid metabolism (44). Recently, fetuin-B has been suggested as a crucial secreted hepatocyte factor linking hepatic steatosis to impaired glucose metabolism (45). Circulating fetuin-B concentrations are significantly elevated in non-alcoholic fatty liver disease (46), hepatic steatosis, type 2 diabetes mellitus (47) and gestational diabetes mellitus (48). In addition, serum fetuin-B levels positively correlate with intrahepatic triglyceride content, providing evidence for a relationship between fetuin-B and dyslipidemia (44). There might be a complex interplay among fetuin-B, metabolic syndrome, and vascular complications, since diabetes and dyslipidemia participate in the pathogenesis of atherosclerosis and cardiovascular diseases. Fetuin-B levels may influence the risk of rupture of atherosclerotic plaques and thereby the risk of acute myocardial infarction (24, 49). High fetuin-B levels are associated with the presence of coronary artery disease and acute coronary syndromes (50). These associations among fetuin-B, atherosclerosis, and cardiovascular diseases suggest the need for further work to clarify the role of fetuin-B in ISR.

APOC3 is an 8.8-kDa glycoprotein composed of 79 amino acid residues, synthesized mainly in the liver. It is a natural lipolysis inhibitor and is closely associated with the metabolism of triglyceride-rich lipoproteins and hepatic clearance of remnant particles (51). Polymorphism of the APOC3 gene appears to influence the risk of hyperlipidemia, and APOC3 concentration and triglyceride levels correlate strongly with risk of coronary artery disease. In mouse models, deleting the APOC3 gene reduces triglyceride levels, while overexpressing APOC3 increases them (52, 53). Several polymorphisms in the human APOC3 gene affect levels of plasma triglycerides, thereby influencing the risk of ischemic vascular disease and severity of coronary heart disease (54, 55). In addition, loss-of-function mutations in APOC3 have been associated with low circulating triglycerides and reduced incidence of cardiovascular disease (56). We observed in the present study that patients with ISR had markedly elevated plasma APOC3 levels, although we cannot explain why. One possible explanation comes from *in vitro* experiments showing that APOC3 can activate atherosclerotic and inflammatory pathways in the vasculature (57–59), and a study of APOC3 in human monocytes showing that it can promote sterile inflammation and organ damage by serving as an endogenous nod-like receptor family pyrin domain-containing 3 activator (60). Whether APOC3 plays the same role in ISR remains to be seen; in any event, we hypothesize that plasma APOC3 levels can provide information about ISR. Further studies should explore whether APOC3 contributes to ISR through multiple vascular mechanisms involving atherosclerosis and proinflammatory responses.

Epidemiological studies have consistently shown that elevated levels of plasma high-density lipoprotein cholesterol (HDL-C) and decreased levels of LDL-C inversely correlate with the incidence of cardiovascular disease (61). Our previous work reported that a high LDL-C/HDL-C ratio was associated with cardiovascular events in patients with acute coronary syndrome after PCI and drug-eluting stent implantation (41). This ratio depends on the levels and activity of CETP, a transport protein involved in the bidirectional exchange of cholesteryl esters from HDL to potentially pro-atherogenic non-HDL fractions. CETP may play a role in atherosclerosis development (62), and we found higher plasma levels of CETP in patients with ISR. The presence of CETP in endothelial cells can generate vascular oxidative stress and induce endothelial dysfunction (63). Overexpression of CETP decreases serum HDL-C levels and increases the accumulation of macrophage-derived foam cells in lesions, thereby contributing to atherosclerosis (64). Indeed, polymorphisms in the CETP gene that affect the levels and activity of the protein alter susceptibility to atherosclerosis (65, 66). Inhibition of CETP reduces LDL-C and increases HDL-C, thereby reducing the risk of atherosclerotic cardiovascular disease (67). Thus, inhibition of CETP is considered a promising therapeutic strategy to raise plasma HDL-C and prevent cardiovascular disease. In animal models, inhibiting CETP activity with anacetrapib can reduce the risk of stent-induced thrombosis and neoatherosclerosis (68). Our results and the literature suggest that CETP concentration may correlate with ISR risk, which may provide exciting leads in future work to clarify the pathogenesis of ISR and develop ways to treat or prevent it.

Our study has a few limitations worth mentioning. First, due to the fact that we have reported 1,696 proteins, and at least one unique peptide was used for protein identification, the results obtained should be interpreted with caution. Second, this study is performed at a relatively small sample size and the number of proteins selected in our verification experiment was also very limited. Targeted proteomics quantification methods such as parallel reaction monitoring or multiple reaction monitoring should be carried out to validate the alteration of the other proteins in a larger sample size. Third, since our results are based mainly on proteomic analysis, in-depth research studies should be conducted to elucidate the underlying biological mechanism of identified proteins in the development of ISR, including experiments in *vivo* and in *vitro*.

## Conclusions

In summary, using TMT coupled with an LC-MS/MS proteomics strategy, we provide the first global plasma protein profile of ISR. Bioinformatic analyses suggest that differentially abundant proteins in ISR patients are involved in different biological processes and pathways, suggesting a variety of mechanisms that may contribute to the ISR. Our results provide insights and identify critical proteins to guide future studies of the molecular mechanisms of ISR, as well as efforts to develop diagnostic biomarkers and therapies.

## Data Availability Statement

The datasets presented in this study have been deposited to the ProteomeXchange Consortium via the iProX partner repository (69). The names of the repository/repositories and accession number(s) can be found below: http://www.proteomexchange.org/, PXD026890.

## Ethics Statement

The studies involving human participants were reviewed and approved by Meizhou People's Hospital. The patients/participants provided their written informed consent to participate in this study. Written informed consent was obtained from the individual(s) for the publication of any potentially identifiable images or data included in this article.

## Author Contributions

JH, SL, and ZZ conceived and designed the study. QD and SL contributed to experiments and bioinformatic analysis. XQ, XD, and WZ provided clinical data and collected the samples. JH, SL, and QD drafted the manuscript. JH and ZZ reviewed the manuscript. All authors contributed to the article and approved the submitted version.

## Funding

This work was supported by grants from the National Natural Science Foundation for Young Scientists of China (82002216 and 82000410), Medical Scientific Research Foundation of Guangdong Province (A2020418 and A2021144), Guangdong Provincial Key Laboratory of Precision Medicine and Clinical Translation Research of Hakka Population (2018B030322003), Science and Technology Program of Meizhou (2019B0202001), and Scientific Research and Cultivation Project of Meizhou People's Hospital (PY-A2019001, PY-C2019013, and PY-C2021010).

## Conflict of Interest

The authors declare that the research was conducted in the absence of any commercial or financial relationships that could be construed as a potential conflict of interest.

## Publisher's Note

All claims expressed in this article are solely those of the authors and do not necessarily represent those of their affiliated organizations, or those of the publisher, the editors and the reviewers. Any product that may be evaluated in this article, or claim that may be made by its manufacturer, is not guaranteed or endorsed by the publisher.

## Supplementary Material

Supplementary Table 1 Overview of the information obtained by tandem mass tag (TMT) quantitative proteomics identification.

Supplementary Table 2 The list of identified peptides.

Supplementary Table 3 The list of identified proteins.

Supplementary Table 4 Detailed information of the proteins identified by tandem mass tag (TMT) quantitative proteomics.

Supplementary Table 5 Differentially abundant proteins between non-ISR patients and HCs.

Supplementary Table 6 Differentially abundant proteins between ISR patients and HCs.

Supplementary Table 7 Differentially abundant proteins between ISR and non-ISR patients.

Supplementary Table 8 Overlap of increased abundance proteins derived from TMT analysis between comparisons of ISR patients with non-ISR patients and of ISR patients with HCs.

Supplementary Table 9 Gene Ontology (GO) analysis of the differentially abundant proteins between ISR and non-ISR patients.

Supplementary Table 10 Enrichment analysis of the differentially abundant proteins between ISR and non-ISR patients.
